# Clinical and laboratory differences between chromosomal and undefined causes of non-obstructive azoospermia: A retrospective study

**DOI:** 10.1590/1516-3180.2022.0281.R1.30082022

**Published:** 2022-11-28

**Authors:** Luísa Riccetto, Tarsis Paiva Vieira, Nilma Lucia Viguetti-Campos, Tais Nitsch Mazzola, Mara Sanches Guaragna, Helena Fabbri-Scallet, Maricilda Palandi de Mello, Antonia Paula Marques-de-Faria, Andrea Trevas Maciel-Guerra, Gil Guerra

**Affiliations:** I Undergraduate Medicine Student, Pontifícia Universidade Católica de Campinas (PUCCAMP), Campinas (SP), Brazil; and Member, Grupo Interdisciplinar de Estudos da Determinação e Diferenciação do Sexo (GIEDDS), Universidade Estadual de Campinas (UNICAMP), Campinas (SP), Brazil.; Undergraduate Medicine Student, Pontifícia Universidade Católica de Campinas (PUCCAMP), Campinas (SP), Brazil; and Member, Grupo Interdisciplinar de Estudos da Determinação e Diferenciação do Sexo (GIEDDS), Universidade Estadual de Campinas (UNICAMP), Campinas (SP), Brazil.; II PhD. Professor, Department of Translational Medicine, School of Medical Sciences, Laboratory of Human Cytogenetics, Universidade Estadual de Campinas (UNICAMP), Campinas (SP), Brazil.; III PhD. Laboratory Worker, Department of Medical Genetics Genomics, School of Medical Sciences, Laboratory of Cytogenetics, Universidade Estadual de Campinas (UNICAMP), Campinas (SP), Brazil.; IV PhD. Laboratory Worker, Laboratory of Human Molecular Genetics, Center of Molecular Biology and Genetic Engineering, Universidade Estadual de Campinas (UNICAMP), Campinas (SP), Brazil.; V PhD. Researcher, Laboratory Worker, Laboratory of Human Molecular Genetics, Center of Molecular Biology and Genetic Engineering, Universidade Estadual de Campinas (UNICAMP), Campinas (SP), Brazil.; VI PhD Researcher, Laboratory Worker, Laboratory of Human Molecular Genetics, Center of Molecular Biology and Genetic Engineering, Universidade Estadual de Campinas (UNICAMP), Campinas (SP), Brazil.; VII PhD. Professor, Laboratory of Human Molecular Genetics, Center of Molecular Biology and Genetic Engineering, Universidade Estadual de Campinas (UNICAMP), Campinas (SP), Brazil.; VIII PhD. Professor, Department of Medical Genetics and Genomics Medicine, School of Medical Sciences, Universidade Estadual de Campinas (UNICAMP); and Member, Grupo Interdisciplinar de Estudos da Determinação e Diferenciação do Sexo (GIEDDS), Universidade Estadual de Campinas (UNICAMP), Campinas (SP), Brazil.; PhD. Professor, Department of Medical Genetics and Genomics Medicine, School of Medical Sciences, Universidade Estadual de Campinas (UNICAMP); and Member, Grupo Interdisciplinar de Estudos da Determinação e Diferenciação do Sexo (GIEDDS), Universidade Estadual de Campinas (UNICAMP), Campinas (SP), Brazil.; IX PhD. Professor, Department of Medical Genetics and Genomics Medicine, School of Medical Sciences, Universidade Estadual de Campinas (UNICAMP); and Member, Grupo Interdisciplinar de Estudos da Determinação e Diferenciação do Sexo (GIEDDS) Universidade Estadual de Campinas (UNICAMP), Campinas (SP), Brazil.; PhD. Professor, Department of Medical Genetics and Genomics Medicine, School of Medical Sciences, Universidade Estadual de Campinas (UNICAMP); and Member, Grupo Interdisciplinar de Estudos da Determinação e Diferenciação do Sexo (GIEDDS) Universidade Estadual de Campinas (UNICAMP), Campinas (SP), Brazil.; X PhD. Professor, Department of Pediatrics, School of Medical Sciences Universidade Estadual de Campinas (UNICAMP); and Member, Grupo Interdisciplinar de Estudos da Determinação e Diferenciação do Sexo (GIEDDS), Universidade Estadual de Campinas (UNICAMP), Campinas (SP), Brazil.; PhD. Professor, Department of Pediatrics, School of Medical Sciences Universidade Estadual de Campinas (UNICAMP); and Member, Grupo Interdisciplinar de Estudos da Determinação e Diferenciação do Sexo (GIEDDS), Universidade Estadual de Campinas (UNICAMP), Campinas (SP), Brazil.

**Keywords:** Sperm count, Infertility, Gonadotropins, Disorders of sex development, Arm span, Height, Sexual development disorders

## Abstract

**BACKGROUND:**

Knowledge of clinical and laboratory differences between chromosomal and undefined causes aids etiological research on non-obstructive azoospermia.

**OBJECTIVE:**

Compare clinical and laboratory differences between men with non-obstructive azoospermia due to chromosomal anomalies versus undefined causes

**DESIGN AND SETTING:**

A cross-sectional retrospective study conducted at a public university hospital in Campinas (Brazil)

**METHODS:**

All men aged 20–40 years with non-obstructive azoospermia were included in the analysis.

**RESULTS:**

The 107 cases included 14 with Klinefelter syndrome (KS) (13%), 1 with mosaic KS, 4 with sex development disorders (2 testicular XX, 1 *NR5A1* gene mutation, and 1 mild androgen insensitivity syndrome) (4%), 9 with other non-obstructive azoospermia etiologies (8%), and 79 with undefined causes. The 22 chromosomal anomaly cases (14 KS, 1 mosaic KS, 2 testicular XX, 4 sex chromosome anomalies, and 1 autosomal anomaly) were compared with the 79 undefined cause cases. The KS group had lower average testicular volume, shorter penile length, and lower total testosterone levels but greater height, arm span, serum luteinizing hormone (LH) and follicle stimulating hormone (FSH) levels, and gynecomastia frequency (absent in the undefined group and affecting more than half of the KS group). Patients with testicular XX DSD had LH, FSH, and penile length data intermediate between the KS and undefined cause groups, testicular volume similar to the KS group, and other data similar to the undefined group.

**CONCLUSION:**

Clinical and laboratory data differentiate men with non-obstructive azoospermia and chromosomal anomalies, particularly KS and testicular XX, from those with undefined causes or other chromosomal anomalies.

## INTRODUCTION

Male infertility is defined as the biological inability of a man to induce pregnancy in a fertile woman after unprotected sexual intercourse for at least one year.^1^ A study of a North American population revealed that 12% of male individuals aged 15–44 years are infertile.^2^ The main factors related to infertility include obesity, infection, neoplasms, cryptorchidism, smoking, varicocele, chromosomal anomalies, sperm duct defects, scrotal exposure to high temperatures, hormonal imbalances, celiac disease, medications, heavy metal poisoning, and exposure to ionizing radiation.^3, 4, 5^

Regarding the genetic causes of male infertility, approximately 15–20% of men with severe non-obstructive azoospermia or oligospermia have microdeletions on the long arm of the Y chromosome (AZFa, b, or c regions) where the spermatogenesis genes are located.^6,7^ Some cases of male infertility may also be related to disorders of sex development (DSD), such as Klinefelter syndrome (KS), testicular 46,XX DSD, and disorders related to the synthesis or action of testicular hormones.^8^

Currently available DSD cohorts in the literature mostly include pediatric patients, with genital ambiguity being the main reason for referral.^9,10^ In contrast, studies of genetic causes of male infertility have mainly focused on chromosomal anomalies and Yq microdeletions.^11,12^ In these studies, as well as in the guidelines on male infertility,^3^ DSD are not specifically considered a cause.

A recent study by our group of 84 men with non-obstructive infertility (azoospermia or severe oligospermia) showed that 10 (12%) had KS, 1 had testicular DSD 46,XX, and 1 had mild androgen insensitivity syndrome (MAIS). The same study also observed 4 cases of structural anomalies of the Y chromosome, 2 Yq microdeletions, and 1 autosomal anomaly corresponding to 22% of the evaluated cases. Of total patients studied, only 14 had increased serum FSH levels, 23 had increased FSH and LH levels, and 13 had decreased testosterone levels.^13^

Considering that about one-fourth of men with severe infertility have chromosomal anomalies and that the clinical and laboratory characteristics of these men, compared to those without chromosomal anomalies, have been insufficiently studied in the literature, it is necessary to verify whether these data can be useful for distinguishing men with chromosomal anomalies from those without a clear cause of their non-obstructive azoospermia.

## OBJECTIVE

To compare clinical and laboratory data of men with non-obstructive azoospermia due to chromosomal anomalies versus undefined causes.

## METHODS

### Population data

This observational cross-sectional retrospective study was based on a medical record analysis. All included patients received medical care from the Interdisciplinary Group for the Study of Sex Determination and Differentiation (Grupo Interdisciplinar de Estudos da Determinação e Diferenciação do Sexo, GIEDDS) at the Hospital das Clínicas da Universidade Estadual de Campinas (HC-UNICAMP), in Campinas, Sao Paulo, Brazil. This study included all men aged 20–40 years with non-obstructive azoospermia (absence of sperm in the ejaculate secondary to impaired spermatogenesis) who were referred to our outpatient clinic for etiological clarification between January 2010 and December 2019. The inclusion criteria were non-obstructive azoospermia (confirmed by at least two sperm counts) and no history of medication use or a disease recognized as a possible cause of male infertility. The exclusion criterion was incomplete clinical and/or laboratory data in the medical records. The project was approved by the Institution's Research Ethics Committee (CAAE: 31480020.0.0000.5404) on June 2, 2020.

### Clinical data

The following clinical data were obtained from the patients' medical records: age (years), auto-declared race (white, brown, black, yellow, other), family history (e.g., parental consanguinity or recurrence of infertility within the family, which was considered positive if there were male relatives as far as third cousins who did not induce spontaneous pregnancies), educational status (illiterate, complete elementary and middle school or 8 years, complete high school or 11 years, or undergraduate school completed or not), height (cm), difference from average family target height,^14^ arm span (cm) and ratio to height, body mass index (BMI) in kg/m^2^, penile length (in cm),^15^ and testicular volume (in mL).

### Laboratory and genetic data

In all cases, a conventional G-banding karyotype study was performed with 400-band resolution and a minimum count of 20 metaphases. In those with normal karyotypes, analysis of a Yq microdeletion was performed using the polymerase chain reaction multiplex technique and 28 molecular markers that mapped the three regions considered the azoospermia locus (AZFa, AZFb, and AZFc).

The following laboratory data were also obtained from the medical records: LH (IU/L), FSH (IU/L), and total testosterone (ng/mL) serum concentrations, karyotype data, Yq microdeletion data, and other cytogenetic or molecular tests.

### Statistical analysis

The statistical analysis was performed using SPSS (Statistical Package for the Social Sciences), version 20.0, Chicago, USA applying absolute and relative frequency data using the Mann-Whitney and Fisher tests with a level of significance of P < 0.05.

## RESULTS

A total of 150 cases were evaluated, but only 107 met the inclusion criteria: 14 with KS, 1 with mosaic KS (47,XXY/46,XY), 4 with other sex chromosome anomalies [1 with 47,XYY; 1 with 46,X,idic(Yq); 1 with 46,X,del(Y)(q12); 1 with 46,X,inv(Y) (p11.2q11.23)], 1 with autosomal anomaly [46,XY,t(6;13) (p12;p13)], 2 with Yq microdeletion, 2 with autosomal recessive infertility with increased FSH and no mutation in the *FSHR* gene, 1 with MAIS and confirmed mutation in the *AR* gene, 2 with testicular 46,XX DSD, 1 with mutation in the *NR5A1* gene, and 79 with undefined cause. In the defined cause group, the cases of KS, mosaic KS, MAIS, *NR5A1* mutation, and testicular XX were considered DSD (i.e., 19 of 107 [17.7%]), with chromosomal anomalies in KS, mosaic KS, testicular XX, and other autosomal or sex chromosome anomalies (22 of 107 [20.6%]). Of these 107 cases, 101 were included in this study, of which 79 had undefined causes and 22 had chromosomal anomalies (Table 1). Patients with a defined cause of non-obstructive azoospermia but no chromosomal abnormalities (2 with Yq microdeletion, 2 with autosomal recessive infertility with increased FSH and no mutation in the *FSHR* gene, 1 with MAIS and confirmed mutation in the *AR* gene, and 1 with mutation in *NR5A1* gene) were excluded. Among the 101 cases, the mean age was 30.4 years (SD: 4.8 years; range, 22–40 years); the auto-declared race was white for 67, brown for 20, and black for 15; and the educational status was illiterate for 11, completed elementary and middle school for 53; completed high school for 27, and undergraduate school for 10. No characteristics (age, race, or educational status) differed between those with KS, mosaic KS, sex chromosome and autosomal anomalies, testicular XX, and undefined etiologies. Obesity was present in 42 patients, although the data did not differ between the groups.

**Table 1.** Clinical and laboratory data of 101 cases (22 with chromosomal abnormalities, 79 with undefined cause) of men with non-obstructive azoospermia

	KS	KS mos	SCA	AA	Testicular XX	Undefined
n	14	1	4	1	2	79
Penile length (cm)	9	9	11	13	10	11
Gynecomastia (n)	8	0	0	0	0	0
Test vol (mL)	4	20	19	20	5	15
H (cm)	179	178	170	176	172	173
PH (cm)	170	172	168	170	168	169
H – PH (cm)	9	6	2	6	4	4
AS (cm)	183	186	174	175	174	177
AS/H	1.02	1.04	1.02	1.0	1.02	1.02
BMI (kg/m^2^)	28.1	28.7	35.9	21.9	23.1	26.2
LH (UI/L)	21.6	3.8	7.3	4.6	11.5	6.8
FSH (UI/L)	30.5	2.8	17.4	1.7	22.0	14.1
Total testosterone (ng/mL)	2.7	4.7	2.2	3.6	5.7	4.5

AA = autosomal anomaly; AS = arm span; BMI = body mass index; FSH = follicle-stimulating hormone; H = height; KS = Klinefelter syndrome; KS mos = mosaic KS; LH = luteinizing hormone; mos = mosaicism; n = number of patients; SCA = sex chromosome abnormality; test vol, mean bilateral testicular volume.

Due to the small number of cases in some of these groups, we decided to compare only the data between the 14 patients with KS and 79 patients in the undefined cause group.

There was no statistically significant difference (Mann-Whitney test) between the KS group (n = 14) and the undefined group (n = 79) in parental height (P = 0.37), arm span and height ratio (P = 0.98), or BMI (P = 0.15) (Table 1). However, a statistically significant difference was observed in mean testicular volume (P < 0.0001), penile length (P < 0.001), and total testosterone level (P < 0.001), all of which were lower in the KS group, as were stature (P < 0.001), difference between the patient;s height and mean parental height (P < 0.0001), arm span (P < 0.01), LH serum concentrations (P < 0.0001), and FSH serum concentrations (P < 0.0001), which were all higher in the KS group (Table 2). A significant difference was observed in the presence of gynecomastia between the KS and undefined groups; it was absent in the undefined cause group and present in more than half of the KS patients (Table 2) (Fisher test, P < 00001). The only other case of gynecomastia was the MAIS patient.

**Table 2.** Clinical and laboratory data of 93 men with non-obstructive azoospermia (14 KS cases, 79 with an undefined etiology)

	KS	Undefined
n	14	79
Penile length (cm)*	9	11
Gynecomastia (n)^#^	8	0
Test vol (mL)*	4	15
H (cm)*	179	173
PH (cm)	170	169
H – PH (cm)*	9	4
AS (cm)*	183	177
AS/H	1.02	1.02
BMI (Kg/m^2^)	28.1	26.2
LH (UI/L)*	21.6	6.8
FSH (UI/L)*	30.5	14.1
Total testosterone (ng/mL)*	2.7	4.5

AS = arm span; BMI = body mass index; FSH = follicle-stimulating hormone; H = height; LH = luteinizing hormone; KS = Klinefelter syndrome; n = number of patients; test vol, average bilateral testicular volume. *Statistically significant difference between the KS and undefined groups (Mann-Whitney test); ^#^statistically significant difference between the KS and undefined groups (Fisher test).

The mosaic KS patient exhibited laboratory data (LH, FSH, and total testosterone) like those of the undefined group as well as average testicular volume, penile length, height, arm span, and BMI, like the KS group (Table 1). Patients with testicular XX DSD (n = 2) had LH, FSH, and penile length data intermediate between the KS and undefined cause groups, testicular volume like the KS group, and other data similar to the undefined group (Table 1). The data for the remaining cases (other sex chromosomes or autosomal anomalies) did not differ significantly from those of the undefined group.

## DISCUSSION

This study determined that approximately 20% of non-obstructive azoospermia cases were associated with chromosomal anomalies or DSD. These results correspond with the percentage reported in the current literature (approximately 15%).^16,17^

KS is the most frequent genetic and chromosomal cause of non-obstructive azoospermia. According to Abramsky and Chapple^18^ (1997), approximately 3% of male infertility cases are caused by KS. In the present study, 15 of 107 cases (14%) of non-obstructive azoospermia were caused by KS. However, it is important to note that only 25% of KS carriers are diagnosed during their lifetime and that most diagnoses occur in adulthood during patient infertility investigations.^19,20^

Individuals with testicular XX can also be identified among patients with non-obstructive azoospermia. For the most part, these individuals have the *SRY* gene translocated on one of the X chromosomes, and their phenotype is virtually identical to that of individuals with KS. In such cases, the lack of sperm production is due to the absence of other genes on the Y chromosome.^21^

Other autosomal or sex chromosome anomalies can also occur but at a much lower frequency, as observed in the present study. Cases of XY partial gonadal dysgenesis, combined gonadal dysgenesis, and ovotesticular DSD can also be found among individuals with typical or highly virilized male genitalia, which is only detected during adulthood infertility investigations.^22^ Other 46,XY DSD that are not associated with gonadal differentiation disorders may also go undiagnosed during childhood and have infertility as their main complaint. This is what often happens in cases of hypogonadotropic hypogonadism, milder forms of testosterone synthesis defects, and also in androgen receptor mutations with a male phenotype (MAIS) or barely noticeable genital ambiguity, in which there is a reduction in sperm production due to defects in androgenic activity.^23,24^ Individuals with 5-alpha-reductase type 2 deficiency may also have more pronounced androgenization of the external genitalia which goes undiagnosed in childhood but present as adulthood infertility due to underdevelopment of the prostate and seminal vesicles.^25^ In the present study, apart from the KS and testicular XX cases, 1 case of XY partial gonadal dysgenesis due to the *NR5A1* gene mutation and 1 case of MAIS with the *AR* gene mutation were diagnosed. Therefore, the results of the present study show that observation of clinical and laboratory data is very important for the differentiation of cases of non-obstructive azoospermia with chromosomal anomalies or DSD of other etiologies.

Except for the KS and testicular XX cases, the other sex chromosomes and autosomal anomalies did not exhibit clinical or laboratory differences from cases of undefined cause. Few studies in the literature have shown these results.^26,27^ However, compared to undefined cases, KS patients presented with smaller testicular volume, shorter penis length, taller stature, greater arm span, higher serum LH and FSH concentrations, and lower testosterone levels. Gynecomastia was absent in the undefined cause group and was present in more than half of the patients with KS. In the literature, testicular volumes lower than 6 mL were present in more than 95% of KS cases,^28^ the same being true for increased serum gonadotropins.^28, 29, 30^ Decreased serum testosterone levels were present in 63–85% of KS cases and occurred more frequently in the older age group.^28, 29, 30^ Gynecomastia is reportedly present in 38–75% of affected adolescents and adults.^26,28,29^ Increased stature occurs in approximately 30% of children and adults with KS.^26,28,31^ Smaller penile size is present in 10–25% of children and adults with KS.^26,28^ Therefore, although the findings described in the present study are frequent in KS, our data confirm that they are an important tool for differentiating patients with KS from those with other non-chromosomal cases of non-obstructive azoospermia.

The patient with mosaic KS had a laboratory profile similar to that of the undefined group but had clinical features similar to KS with the exception of normal testicular volume. Previous studies demonstrated that it is difficult to pinpoint a specific clinical feature in cases of sex chromosome mosaicism without actually knowing the percentage of each lineage in various tissues, including gonads.^32^

Patients with testicular XX DSD (n = 2) exhibited clinical features similar to those of the undefined group except for decreased testicular volume and hypergonadotropic hypogonadism, but these were not as severe as the results seen in the KS group. Similar data have been described in the literature.^21,33,34^

## CONCLUSION

Despite the fact that this study has all the limitations of retrospective data collection, it provides important clinical information that supports medical investigations in men with non-obstructive infertility from a significant sample evaluated homogeneously by a single clinical service over a 10-year period with karyotyping performed in all cases and Yq microdeletions investigated in all patients with a normal karyotype.

Therefore, two main conclusions may be drawn from this study: first, chromosomal anomalies were the cause of approximately 20% of non-obstructive azoospermia cases. Second, clinical and laboratory differences existed among different non-obstructive azoospermia etiologies, especially undefined, KS, and testicular XX cases. For this reason, the results of this study provide an important information resource that will be very useful for physicians and other healthcare professionals during investigations and requests for complementary tests during the etiological evaluation of non-obstructive azoospermia.
