# Erratum

**DOI:** 10.1590/1516-3180.2022.0281.R1.300820221erratum

**Published:** 2023-01-30

In the manuscript titled “Clinical and laboratory differences between chromosomal and undefined causes of non-obstructive azoospermia: A retrospective study”, DOI number 10.1590/1516-3180.2022.0281.R1.30082022, published in the Sao Paulo Medical Journal - Epub ahead of print:

**Where it read:**

“Grupo Interdisciplinar de Estudos da Determinação e Diferenciação do Sexo (GIEDDS), Hospital das Clínicas da Universidade Estadual de São Paulo (UNICAMP), Campinas (SP), Brazil.”

**It should read:**

“Grupo Interdisciplinar de Estudos da Determinação e Diferenciação do Sexo (GIEDDS), Hospital das Clínicas da Universidade Estadual de Campinas (UNICAMP), Campinas (SP), Brazil.”

